# Ensemble neuronal responses in a large-scale realistic model of the cerebellar cortex

**DOI:** 10.1186/1471-2202-14-S1-P82

**Published:** 2013-07-08

**Authors:** Sergio Solinas, Timoteo Colnaghi, Egidio D'Angelo

**Affiliations:** 1 Brain Connectivity Center, Istituto Neurologico IRCCS C. Mondino, Via Mondino 2, I-27100 Pavia, Italy; 2 Department of Brain and Behavioural Science, Neurophysiology and Neurocomputation Unit, University of Pavia, Via Forlanini 6, I-27100, Pavia, Italy

## 

Realistic simulation of central networks remains a challenge due to the complexity of internal connectivity and cellular mechanisms involved. We have recently built a realistic model of the cerebellar granular layer [1], which has now been extended to include the molecular layer and Purkinje cells (PC). The model is built in NEURON-PYTHON and is fully scalable, thus allowing to simulate large-scale networks of arbitrary size. The model is made of conductance-based multicompartmental neurons and incorporates dynamic synaptic mechanisms. The model accounts for the principal neuronal types (granule and Golgi cells in the granular layer; Purkinje cells and stellate cells in the molecular layer) and for their density and connectivity (including gap-junctions between Golgi cell dendrites). As in the real network, mossy fiber (mf) branching gives origin to clusters of glomeruli aligned along the parasaggittal plane. The functionality of this cortical cerebellar network model was validated using input patterns, whose impact has been demonstrated previously. (1) Low frequency random mf inputs, simulating background resting activity, induced low frequency oscillations in the granular layer. (2) High frequency bursts delivered to specific mf bundles activated multiple activity spots within the granular layer, with a center-surround configuration. (3) Collision of inputs from multiple active mf bundles generated either coincident excitation or inhibition [2]. (4) Low-frequency MF bursts were reliably transmitted through the granular layer toward PCs aligned along the vertical axis as well as to PCs laying along the parallel fiber axis. Conversely, high-frequency bursts were strongly amplified and transmitted toward PC aligned along the vertical axis but not along the parallel fiber axis[3]. These results, by closely matching experimental recordings, suggest that the model network can be further used to investigate the mechanisms of cerebellar functioning.
